# An open-label, pilot study of veliparib and lapatinib in patients with metastatic, triple-negative breast cancer

**DOI:** 10.1186/s13058-021-01408-9

**Published:** 2021-03-04

**Authors:** Erica M. Stringer-Reasor, Jori E. May, Eva Olariu, Valerie Caterinicchia, Yufeng Li, Dongquan Chen, Deborah L. Della Manna, Gabrielle B. Rocque, Christos Vaklavas, Carla I. Falkson, Lisle M. Nabell, Edward P. Acosta, Andres Forero-Torres, Eddy S. Yang

**Affiliations:** 1 Department of Medicine, Division of Hematology Oncology, University of Alabama at Birmingham, Birmingham, AL USA; 2 Department of Medicine, Brookwood Baptist Health, Birmingham, AL USA; 3 Department of Pharmacology/Toxicology, University of Alabama at Birmingham, Birmingham, AL USA; 4 Department of Radiation Oncology, University of Alabama at Birmingham, Birmingham, AL USA; 5 O’Neal Comprehensive Cancer Center, University of Alabama at Birmingham, 1700 6th Avenue South, HSROC Suite 2232 (176F), Birmingham, AL 35249 USA

**Keywords:** PARP inhibitors, DNA repair, Synthetic lethality, Targeted therapy, Triple-negative breast cancer

## Abstract

**Background:**

Poly (ADP-ribose)-polymerase inhibitors (PARPi) have been approved for cancer patients with germline *BRCA1/2* (g*BRCA1/2*) mutations, and efforts to expand the utility of PARPi beyond *BRCA1/2* are ongoing. In preclinical models of triple-negative breast cancer (TNBC) with intact DNA repair, we have previously shown an induced synthetic lethality with combined EGFR inhibition and PARPi. Here, we report the safety and clinical activity of lapatinib and veliparib in patients with metastatic TNBC.

**Methods:**

A first-in-human, pilot study of lapatinib and veliparib was conducted in metastatic TNBC (NCT02158507). The primary endpoint was safety and tolerability. Secondary endpoints were objective response rates and pharmacokinetic evaluation. Gene expression analysis of pre-treatment tumor biopsies was performed. Key eligibility included TNBC patients with measurable disease and prior anthracycline-based and taxane chemotherapy. Patients with g*BRCA1/2* mutations were excluded.

**Results:**

Twenty patients were enrolled, of which 17 were evaluable for response. The median number of prior therapies in the metastatic setting was 1 (range 0–2). Fifty percent of patients were Caucasian, 45% African–American, and 5% Hispanic. Of evaluable patients, 4 demonstrated a partial response and 2 had stable disease. There were no dose-limiting toxicities. Most AEs were limited to grade 1 or 2 and no drug–drug interactions noted. Exploratory gene expression analysis suggested baseline DNA repair pathway score was lower and baseline immunogenicity was higher in the responders compared to non-responders.

**Conclusions:**

Lapatinib plus veliparib therapy has a manageable safety profile and promising antitumor activity in advanced TNBC. Further investigation of dual therapy with EGFR inhibition and PARP inhibition is needed.

**Trial registration:**

ClinicalTrials.gov, NCT02158507. Registered on 12 September 2014

**Supplementary Information:**

The online version contains supplementary material available at 10.1186/s13058-021-01408-9.

## Translational relevance

Efforts to expand the utility of PARPi beyond *BRCA1/2* have been ongoing, such as inducing a BRCA-like phenotype to create synthetic lethality with PARPi. Our previous preclinical work revealed EGFR inhibition suppressed DNA repair and enhanced sensitivity of *BRCA1/2* wild-type tumors to PARPi. In this study, we translated this into a first-in-human clinical trial and showed that combined EGFR/PARP inhibition with lapatinib and veliparib was safe and had promising antitumor activity. Molecular profiling revealed potential biomarkers of response involving the DNA repair and immune pathways. These results emphasize the value of efforts to increase the number of patients who may benefit from PARP inhibitor combinations.

## Introduction

Poly (ADP-ribose)-polymerase-1 (PARP1) is a nuclear enzyme that recognizes DNA damage and facilitates DNA repair [1, 2]. PARP inhibitors have recently been FDA approved for breast cancer patients with germline mutations in DNA repair genes, specifically those with deleterious BRCA1 and BRCA2 mutations, which constitutes 3–4% of all women with breast cancer and includes 10 to 20% of those with triple-negative breast cancer [3–10]. The role of PARP inhibitors in the setting of non-BRCA-associated cancers has been limited, and efforts to uncover new contextual synthetic lethality have been undertaken.

One potential approach to sensitize non-BRCA cancers to PARP inhibition is to combine with agents that modulate the DNA damage repair pathway. We and others have previously demonstrated that the epidermal growth factor receptor (EGFR) is involved in regulating DNA damage response independent of RAS or p53 status [11–14]. EGFR is overexpressed or amplified in 45–70% of triple-negative breast cancer (TNBC) and is associated with an aggressive disease phenotype [15–17]. Furthermore, overexpression of EGFR is correlated with increased tumor size, lymph node involvement, and decreased survival in invasive breast cancers [15–17]. We demonstrated that lapatinib, an oral dual EGFR/HER2 tyrosine kinase inhibitor, attenuated homologous recombination repair in TNBC cell lines by reducing EGFR-BRCA1 interaction [14]. Importantly, lapatinib induced a contextual synthetic lethality with the PARP inhibitor veliparib in preclinical xenograft models of TNBC.

Based on prior preclinical data demonstrating that EGFR inhibitors can sensitize non-BRCA tumors to PARP inhibitors and lead to an increase in tumor cell death in TNBC models, we performed an open-label pilot study of lapatinib and veliparib combination therapy in patients with refractory TNBC without a BRCA1/2 alteration. Herein, we report the safety/tolerability profile, pharmacokinetics (PK), and antitumor effects of the combination treatment. We also report exploratory gene expression analysis from tumor core biopsy samples prior to treatment in order to compare patients who were partial responders (PR) to those with progressive disease (PD).

## Patients and methods

### Study design and treatment

We conducted a single institution, open-label pilot trial in which eligible patients received veliparib and lapatinib for the treatment of metastatic TNBC. The primary objective was patient safety and tolerability. This study was conducted at The University of Alabama at Birmingham (Birmingham, AL) and was approved by the UAB Institutional Review Board. Written informed consent was obtained from patients prior to any study procedure. The study was registered on ClinicalTrials.gov as NCT02158507.

### Eligibility criteria

Patients with biopsy-proven, metastatic or locally advanced, unresectable TNBC (ER negative, PR negative, HER-2- Neu negative by IHC or FISH) breast cancer (BC) were eligible for participation. Additional eligibility requirements included age ≥ 19 years, ECOG PS of 0–2, adequate organ and marrow function as defined by the study protocol, a left ventricular ejection fraction ≥ 50%, and measurable disease per RECIST criteria v1.1. Negative serum or urine beta-HCG pregnancy test was necessary for patients of child-bearing ages, in addition to an agreement to use an effective means of contraception in this population. Patients were required to have had prior anthracycline and taxane use in the neoadjuvant, adjuvant, or metastatic setting. Patients with more than two prior regimens for metastatic disease were ineligible. Although not mandatory for study entry, a biopsy was requested in patients with reasonably accessible lesions.

All study participants received genetic counseling and had germline testing if indicated as part of the standard of care. No pathogenic germline alterations were found in DNA repair genes. Two participants did not meet guidelines for genetic testing and therefore had low likelihood of harboring germline alterations. Furthermore, this study enrolled patients from 2014 to 2017, a time in which NGS testing was not standard of care at our institution. Therefore, tumor mutation status was not available at the time of enrollment.

### Exclusion criteria

Patients were excluded if they had a history of hypersensitivity to veliparib, lapatinib, or drugs of similar chemical composition. Concomitant investigational or commercial agents or therapies administered with the intent to treat the patient’s malignancy were not allowed, with the exception of bisphosphonates and RANKL inhibitors for bone metastases. Patients with germline BRCA1 or BRCA2 mutations, prolonged QTc interval > 470 ms, and untreated or uncontrolled brain metastases were excluded. In the presence of brain metastases, patients must have had at least one site of measurable disease outside of the central nervous system. Those with prior invasive malignant disease within 5 years (with the exception of in situ skin or cervical cancers), history of HIV, or hepatitis B were excluded.

### Drug supply

Veliparib was provided by AbbVie and lapatinib was supplied by Novartis Pharmaceuticals Corporation.

### Study treatment

Within 4 weeks prior to initiation of therapy, patients underwent baseline clinical evaluation, including medical history, physical exam, performance status, laboratory evaluation, radiographic tumor measurement, EKG, and echocardiogram. Patients with an accessible metastatic site underwent a biopsy.

**Fig. 1:**
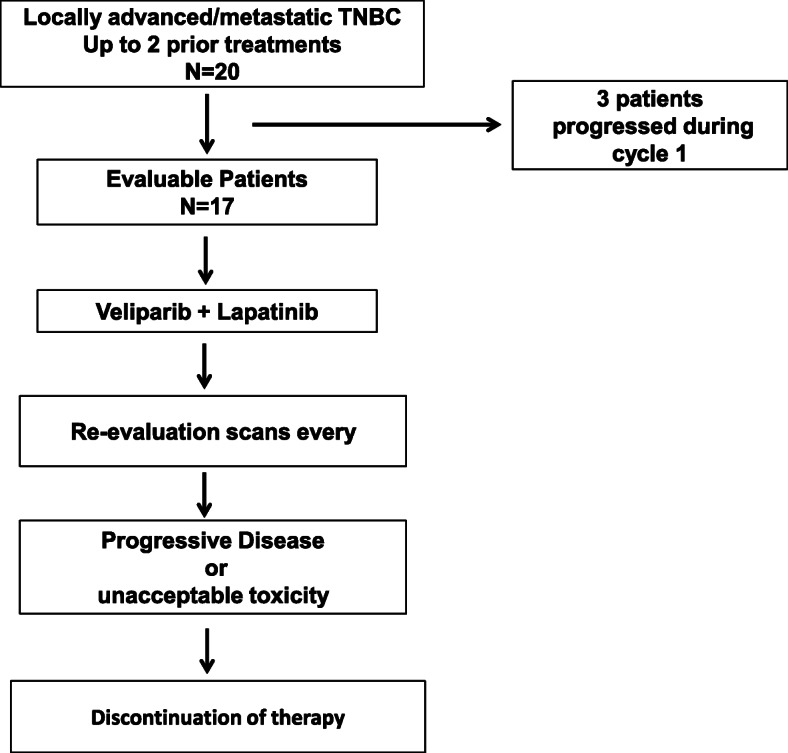
Consort diagram

Twenty patients with evaluable disease were initially enrolled and treated with lapatinib 1250 mg a day by mouth continuously for 28 days, starting at cycle 1 day 1, in combination with veliparib 200 mg every 12 h by mouth for 28 consecutive days, starting on cycle 1 day 2. A cycle of therapy was defined as 28 days. Treatment was administered on an outpatient basis (Fig. 1). Treatment continued in patients with complete response (CR), partial response (PR), or stable disease (SD) until progression of the disease or unacceptable toxicity. SD was defined per protocol as no evidence of progressive disease for > 2 cycles.

### Assessments

Patients were evaluated for response every 2 courses (every 8 weeks) using CT scans according to the RECIST criteria version 1.1. An EKG was obtained on day 1 of each cycle and an echocardiogram was obtained every 3 months. Patients received physical and laboratory evaluation on day 1 of each cycle. AEs were recorded at every visit and graded according to the National Cancer Institute Common Terminology Criteria of Adverse Events (NCI-CTCAE) version 4.03.

### Efficacy and safety evaluation

A continuous safety assessment was utilized to determine the probability of stopping treatment early. There was a high probability of stopping early if the toxicity rate was unacceptably high (i.e., ≥ 60%), with a low probability of stopping early if the toxicity rate was acceptable, i.e., ≤ 30% by Pocock-type boundary [18]. The starting dose of lapatinib was 1250 mg by mouth daily and veliparib 200 mg by mouth twice daily with plans to de-escalate if toxicities were noted. Toxicities were assessed during day 1 of each cycle of therapy according to the NCI Common Toxicity Criteria, version 4.03. Adverse events were assessed among all enrolled patients for the purpose of dose de-escalation decisions.

### Pharmacokinetics and pharmacodynamics assessment

Plasma samples were collected for the bioanalysis of lapatinib to investigate potential interactions between lapatinib and veliparib. The dosing schema (lapatinib on day 1, veliparib on day 2) was designed to understand the PK interactions between the drugs as well as to induce a double-stranded break (DSB) repair defect by administering lapatinib first. PK profiles were evaluated once the steady state of both drugs was reached (day 28).

Blood samples (3 mL, collected in lithium heparin tubes) were taken at predose (0) and then 2, 4, 6, 8, and 24 h after dosing for lapatinib. A validated liquid chromatographic mass spectrometric assay was used to measure lapatinib and veliparib in plasma. Standard PK variables were calculated by a non-compartmental method including maximal plasma or serum concentration (*C*_max_), area under the curve to the last collection point (AUC_last_), area under the curve for dose interval (AUC_0–*t*_), area under the curve extrapolated to infinity (AUC_inf_), time of maximal concentration (*T*_max_), elimination rate constant (*k*_el_), terminal half-life (*t*_1/2_), total clearance (CL), and a volume of distribution (*V*_ss_), using the Phoenix WinNonlin 8.0 (Certara, Princeton, NJ) software. The linearity of PK parameters was explored. Descriptive statistics of the non-compartmental parameters were calculated for cycle 1 and cycle 2.

### Gene expression of tumor core biopsies

Tumor core biopsies were snap-frozen in the procedure room prior to treatment and placed in TRIzol reagent immediately before thawing. Total RNA was isolated using the PureLink RNA Mini kit with TRIzol protocol (Invitrogen/Thermo Fisher, Waltham, MA) and concentrated as needed using the Zymo RNA Clean and Concentrator kit (Irvine, CA). The Breast IO360 panel from NanoString Technologies (Seattle, WA) was run using 100 ng of RNA and counted at the maximum FOV setting, as suggested by the manufacturer. This panel of 776 genes across 23 key breast cancer pathways and processes provides a unique 360-degree view of gene expression for the breast tumor microenvironment and immune response. Standards supplied with the kit were also run in order for further analysis to be performed by NanoString Technologies. This analysis included PAM50 breast cancer subtyping, Tumor Inflammation Signatures (TIS) scores, tumor mutation susceptibility (BRCAness, Homologous Recombination Repair Status, HRD), rate of recurrence (ROR), and other signatures (i.e., Claudin-low) predicting tumor sensitivity to immune attack.

### Statistical method

The primary endpoint of the trial was the safety of the combination of lapatinib and veliparib. Objective response rate (ORR) and progression-free survival (PFS) were secondary endpoints. A total of 20 patients were planned. The sample size was not determined by the statistical power but by the feasibility and relative precision of estimate. Assuming the acceptable toxicity rate with combination therapy was no more than 30%, a two-sided 95% exact confidence intervals (Clopper–Pearson intervals) of toxicity would be estimated from 11.8 to 54.3% with standards error of the estimation less than or equal to 10%. A continuous assessment of toxicity and stopping rules were implemented using the method of Ivanova et al. [18].

The primary analysis of safety was assessed in all patients who received at least one dose of lapatinib and/or veliparib. Descriptive statistics were presented for both quantitative safety data and response rate. During the study, adverse event and serious adverse event (AE/SAE) reporting was summarized by relationship to study drug and intensity. The number and proportion of AE, SAE, and grade 3 or 4 lab toxicities were reported by the body system. Mean, median, and range were used to describe continuous demographic variables and EGFR expression. Frequency and proportion were used to describe categorical variables. At the completion of the study, the secondary endpoint of ORR rate was estimated along with two-sided 95% confidential intervals with the exact method of Clopper–Pearson intervals.

For the gene expression analysis, the nSolver program (4.0) was used to directly compare the counts obtained with the PR samples with the PD samples. Within this program, advanced analysis (2.0) was used to determine the best housekeeping genes to utilize for normalization purposes (using the gnorm algorithm), as well as to determine the genes with the most significant changes (differential expression or DE results, *p* values < 0.05) between the two groups. The fold changes described are the normalized weighted differences reported by nSolver. We also performed IPA analysis using the DEGs generated from Nanostring and Fisher’s exact test for top potential pathways.

## Results

### Patient and treatment

From August 2014 to September 2017, twenty patients were enrolled; seventeen were evaluable for response by RECIST; and 3 patients experienced clinical progression of disease during cycle 1. Evaluable patients completed at least two cycles of therapy. Patient demographics and disease characteristics are shown in Table 1. Of note, 43% of evaluable patients were African–American. Approximately 46% of the study population had prior systemic therapy for metastatic disease. The median number of prior therapies for advanced breast cancer was 1 (range 0–2). Patients with more than 2 prior lines of chemotherapy in the metastatic setting were excluded.

**Table 1:** Patient demographics

Characteristic	*N* = 17
Age, mean (range) years	50 (37–63)
Female	17 (100%)
Race	
Caucasian	9 (57%)
African–American	8 (43%)
No. prior lines of systemic therapy	
0	9
1	4
2	4
Site of recurrence	
Locoregional	6
Distant disease (bone, liver, lung, or brain)	11

### Safety and tolerability

In this phase 1 study, the combination of lapatinib with veliparib did not result in unexpected toxicities. The MTD of the combination was defined at lapatinib 1250 mg QD (days 1–28) with veliparib 200 mg PO twice daily. No dose level reductions were required. Toxicities related to study drugs were limited to grade 1 or 2. Only 2 patients experienced grade 3 toxicities of headache and abdominal pain, respectively, which were attributed to progression of the disease and not due to study drugs. Most common adverse events in decreasing order of frequency included fatigue (6.4%), diarrhea (5.1%), constipation (5.5%), insomnia (4.5%), vomiting (2.9%), anemia (2.6%), headache (2.6%), dizziness (2.6%), dyspnea (2.3%), and rash (2.3%) (Table 2). All treatment-related adverse events were manageable and did not result in any dose reductions, delays, or discontinuations.

**Table 2:** Percentage of treatment-related adverse events

Adverse event	Percentage
Fatigue	6.4
Diarrhea	5.1
Constipation	4.5
Insomnia	4.5
Vomiting	2.9
Anemia	2.6
Headache	2.6
Dizziness	2.3
Dyspnea	2.3
Rash	2.3

### Pharmacokinetics

Seventeen patients underwent lapatinib pharmacokinetic analysis. One patient missed a PK evaluation during cycle 2. The maximum tolerated dose of lapatinib was 1250 mg by mouth daily when in combination with veliparib. Lapatinib pharmacokinetic parameters are described in Supplementary Table S1 in Additional file 1. Lapatinib PK parameters demonstrated moderate interpatient variability during cycle 1. At steady state, lapatinib plasma concentrations were consistent with historical data published on lapatinib monotherapy (Supplementary Figure S1 in Additional file 1) [19, 20]. Co-administration of veliparib did not influence lapatinib PK parameters.

### Antitumor activity

**Fig. 2:**
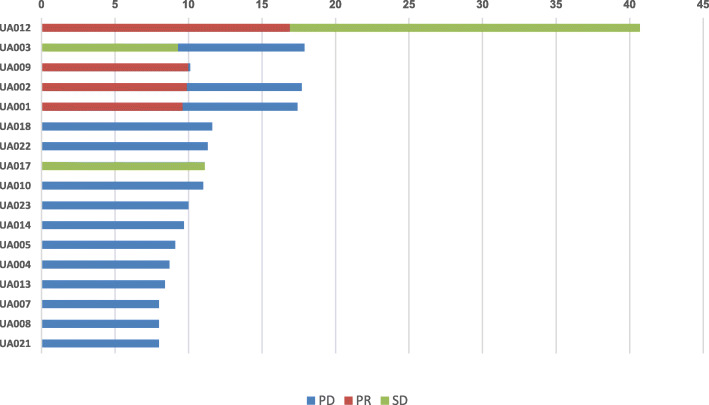
Summary of best overall response by RECIST criteria. Abbreviations: PR, partial response; SD, stable disease; PD, progressive disease

**Fig. 3:**
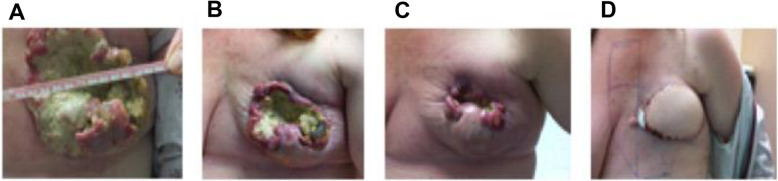
Response in UAB009: **a** pre-treatment and post-treatment at **b** 2 weeks, **c** 2 months, and **d** 5 months

Of the 20 patients enrolled in the study, 17 were evaluated for clinical response. The summary of objective response by RECIST criteria v 1.1 is depicted in Fig. 2. Four patients achieved a partial response (PR), 2 patients had stable disease (SD), and 11 patients had progressive disease (PD). Patient 012 had a prolonged PR of over 10 months. Patient 009 achieved a favorable reduction in disease burden after 2 cycles and was rendered resectable by the combination treatment. The patient discontinued the study to have a left radical mastectomy with reconstruction followed by radiation (Fig. 3). At the time of this manuscript, the patient remains disease free for more than 5 years as of her last clinical visit of July 2020.

### Biomarker analysis

**Fig. 4:**
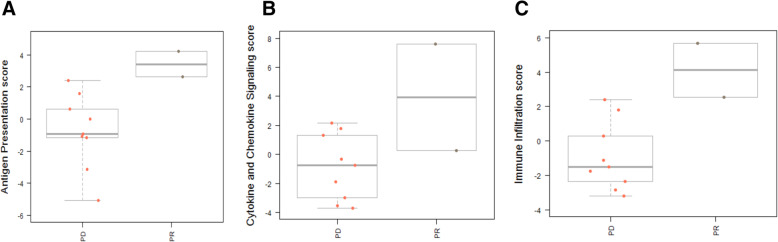
Pre-treatment immune pathway scores in patients with partial response (PR) and progressive disease (PD). **a** Antigen presentation score. **b** Cytokine and chemokine signaling score. **c** Immune infiltration score

Of the 17 patients evaluated for clinical response, tumor biopsies were performed for 11 patients prior to study treatment. The sites from which the biopsies were obtained are listed in Supplementary Table S2. For the remaining patients, the procedural biopsies were determined unsafe due to tumor location. We performed Nanostring analysis on the available samples using the Breast360 panel, which contains 48 published signatures of breast cancer tumor biology, including PAM50, Tumor Inflammation Signature (TIS), and risk of recurrence (ROR)/genomic risk scores. Direct comparison of the 2 PR samples (patients 002, 009) with 9 PD samples (patients 004, 005, 007, 008, 010, 013, 014, 022, 023) showed a significant increase in the expression of genes involved in antigen presentation (HLA-DOB (5.0-fold), HLA-DPA1 (4.6-fold), HLA-DRA (3.7-fold), HLA-DMB (3.5-fold)), immune infiltration (CCL5 (5.5-fold), GZMA (4.8-fold), CD8A (3.3-fold), PDCD1ILG2 (3.3-fold), STAT1 (3.0-fold), LAG3 (2.9-fold)), and cytokine and chemokine signaling (CCR5 (3.6-fold), NOD2 (2.9-fold), CCL8 (2.8-fold), CCL4 (2.7-fold), CCL2 (2.7-fold)) (Fig. 4). Additionally, the TIS trended higher in the responders. The TIS scores were 7.6 and 8.8 for the PR samples and 5.2, 6.1, 7.3, 6.0, 6.1, 7.7, 6.0, 6.3, and 5.9 for the PD samples (Supplementary Figure S2 in Additional file 1). These data suggest the potential of pre-therapy immune status to correlate with response.

To verify the advanced analysis performed via the Nanostring platform, we performed analysis using the DEGs generated from Nanostring and Fisher’s exact test for top potential pathways involved by these DEGs between responders vs. non-responders. Similar to the Nanostring advanced analysis, the top canonical pathways differentially regulated as listed in Supplementary Table 3 (Additional file 1) are involved in immune regulation. Specifically, these include Th1, Th2, T cell exhaustion, neuroinflammation, and MSP-RON signaling in macrophages pathways (Supplementary Table 3 in Additional file 1). For detailed results of the full analysis, refer to Additional files 2 and 3. However, these are preliminary and hypothesis generating, due to the limitations of small sample size and limited number of DEGs.

Interestingly, while all 11 samples showed a “BRCAness” phenotype, the PR samples showed a significant reduction in the DNA damage repair genes BRCA1, CRYAB, and CKB genes (4.8-, 4.5-, and 4.4-fold, respectively), as well as a significant change in genes related to other pathways implicated in DNA damage response (Table 3). Specifically, CALML5, MMP7, COLL11A1, RASGRF1, PTCH1, COL27A1, FGFR2, and NOTCH1 all showed decreased expression of at least 2-fold in PR samples, while PLA2G3, LFNG, PIK3R3, SOCS1, GJB2, STAT1, SOCS2, and HGF all showed at least a 2-fold increase in gene expression. A reduction in genes associated with triple-negative biology across the board was also observed (GABRP (19.9-fold), PROM1 (6.9-fold), HBB (5.7-fold), PDE9A (3.9-fold), BCL11A (3.6-fold), data not shown). Indeed, when PAM50 criteria were used, all the samples were classified as basal-like subtype except for one of the PR samples (002) which was HER2 enriched, and one of the PD samples (patient 008) which was luminal A. All of the breast cancer 360 biological signatures are depicted in Supplementary Figure S2 in Additional file 1 except the risk of recurrence (ROR) scores which were as follows: PR samples 82 (patient 002), 72 (patient 009); PD samples 66 (patient 004), 56 (patient 005), 56 (patient 007), 53 (patient 008), 55 (patient 010), 74 (patient 013), 69 (patient 014), 58 (patient 022), and 74 (patient 023).

**Table 3:** NanoString counts of individual genes of significance (*p* values < 0.05), analyzed from tumor core biopsy samples prior to study treatment. Fold change is PR vs PD (baseline)

Gene name	PR	PD	Fold change	Affected pathway
CALML5	143.27	2255.68	− 15.74	MAPK
MMP7	453.82	5649.84	− 12.45	WNT
BRCA1	57.16	275.57	− 4.82	DNA damage repair, PI3K
PLA2G3	196.18	41.35	4.74	MAPK
CRYAB	568.51	2587.04	− 4.55	DNA damage repair
COL11A1	65.8	294.98	− 4.48	PI3K
CKB	100.47	438.12	− 4.36	DNA
LFNG	722.24	175.07	4.13	Notch
RASGRF1	41.19	156.18	− 3.79	MAPK
PIK3R3	2009.36	558.36	3.6	JAK/STAT, MAPK, PI3K
SOCS1	362.96	104.85	3.46	JAK/STAT
GJB2	395.9	125.75	3.15	DNA damage repair
PTCH1	23.77	72.6	− 3.05	Hedgehog
STAT1	5748.18	1940.41	2.96	JAK/STAT
COL27A1	81.66	209.94	− 2.57	PI3K
SOCS2	498.48	197	2.53	JAK/STAT
FGFR2	133.74	333.34	− 2.49	MAPK, PI3K
NOTCH1	194.05	439.53	− 2.27	Notch
HGF	181.46	81.94	2.21	MAPK, PI3K

## Discussion

This trial investigated the hypothesis that a PARP inhibitor and an EGFR inhibitor are synthetically lethal in BRCA wild-type metastatic TNBC*.* The MTD of the combination was determined at lapatinib 1250 mg QD (days 1–28) with veliparib 200 mg PO twice daily. These results are reasonable as the recommended dose of lapatinib is 1250 mg daily when combined with capecitabine in prior studies [21]. Of the 20 patients included in the study, 6 had a clinical response. One patient had a prolonged PR of over 10 months while another had a rapid response that led to her tumor becoming resectable. At the time of this publication, she remains disease free 5 years after completion of surgery.

There have been several studies targeting EGFR with anti-EGFR monoclonal antibodies such as cetuximab or utilizing EGFR tyrosine kinase inhibitors such as gefitinib or erlotinib with or without chemotherapy in metastatic breast cancer [22]. These studies demonstrated modest response rates with no effect on PFS or OS in TNBC. The lack of clinical efficacy is hypothesized to be due to the molecular biology of TNBC which often harbors RAS and PTEN (30%) mutations which can activate signaling downstream of EGFR [23, 24]. Compensatory upregulation of other receptor tyrosine kinases, such as HER2, accounting for resistance to targeting EGFR [25–27] is also implicated. Blocking EGFR alone is a difficult target to effectively attack in TNBC.

In this study, we chose the oral tyrosine kinase inhibitor lapatinib, which blocks the intracellular domain of EGFR and dual HER1/HER2 receptors [28]. The PARP inhibitor veliparib, which has modest PARP trapping activity [29], was chosen so that any activity observed with the combination in the BRCA1/2 wild-type setting would be more likely due to the combination rather than veliparib or lapatinib alone. Our group and others have shown that EGFR signaling plays a role in regulating the DNA damage response; this mechanism does not depend on wild-type p53 or ras status [14]. In vitro and in vivo models revealed that lapatinib inhibits EGFR/HER2 by attenuating DNA repair maybe by altering protein–protein interactions between EGFR and BRCA1 [14]. Importantly, because EGFR inhibition attenuated homologous recombination repair, we hypothesized that lapatinib could induce a synthetic lethal interaction with the PARP inhibitor veliparib, which targets HR defective cells to promote apoptosis in TNBC cells. These preclinical data demonstrated suppression of DNA repair with EGFR inhibition and thus supported expanding clinical utility of PARP inhibitors beyond BRCA. This was the first-in-human clinical trial combining lapatinib with veliparib in TNBC.

Lapatinib is metabolized extensively through the liver and is a strong CYP3A4/5 substrate with minimum metabolism through CYP2C8, 2C19, and with negligible renal excretion [20]. Its oral absorption is variable with steady state achieved at 6–7 days with a true elimination half-life of 24 h [20, 30]. As a single agent, the most frequent adverse effects of lapatinib include gastrointestinal toxicity (i.e., nausea, vomiting, diarrhea), hepatotoxicity, neutropenia, and decline in left ventricular ejection fraction [31]. Conversely, veliparib is not a potent inhibitor, nor an inducer, of the major human cytochrome P450s (CYPs), suggesting a minimal potential for drug–drug interactions (DDIs) at the anticipated therapeutic concentrations [32, 33]. With veliparib, the most commonly reported adverse events are nausea, fatigue, anemia, diarrhea, and decreased appetite [34]. As expected, there were no drug interactions observed in our study as plasma concentrations of lapatinib appeared to be unaffected by the presence of veliparib. In fact, PK data was similar to previously published data on the pharmacokinetics of lapatinib [19, 20].

In our combination trial, adverse events were modest and limited to grade 1 or 2 level toxicities in less than 10% of the studied population. No DLTs were noted nor was early termination of drug administration required during this study. There are overlapping toxicities known in both the PARP inhibitors and EGFR inhibitor class drugs, which include nausea, fatigue, vomiting, diarrhea, and headache. The low frequency and severity of AEs was somewhat surprising. It has been described that lapatinib in combination with chemotherapy (i.e., gemcitabine) has shown to have GI toxicities of nausea, vomiting, and increased liver function test that range from 50 to 70% [19]. We believe that the anti-inflammatory effects of the PARP inhibitor veliparib may have reduced the GI toxicities in the combination. Anti-inflammatory effects of veliparib have been described in traumatic brain injuries in animal models when utilized up to 24 h after the event [35]. Furthermore, PARP inhibitors have demonstrated effects on reducing pro-inflammatory mediators in the plasma such as TNF-α, IL-1β, IL-2, IL-4, IL-6, IL-12, IP-10, and KC on in vitro models of pancreatitis as well as in vivo models of wound healing injuries [36, 37]. Of note, veliparib is the only PARP inhibitor that has been successfully combined with chemotherapy, including the recent BROCADE3 trial [38].

One of the limitations of our study was that tissue biopsies were not mandatory and with the small sample size, it is difficult to draw conclusions on exploratory inflammatory markers in the plasma or tissue samples. However, we were able to utilize Nanostring and the Breast IO360 gene expression analysis of pre-treatment tumor core biopsies to explore potential biomarkers in the responders vs. non-responders.

Since this panel provides a unique 360-degree view of cancer-pathway gene expression for the breast tumor microenvironment and immune response, it was interesting to note that the responders initially showed significant increases in genes involved with baseline antigen presentation, immune filtration, and cytokine and chemokine signaling. The high Tumor Inflammation Signature (TIS) in the responders supports this finding. This signature has been previously reported to predict responses to anti-PD1 therapy and determine hot and cold immune status. These results corroborate with recent data supporting the combination of PARP inhibitors with checkpoint inhibitors [39]. By inducing genomic instability with PARP inhibition, this could generate neoantigens, stimulate interferon signaling, and activate other immune pathways [40]. While it is possible that patients could have responded to PARPi alone in the setting of an activated immune response, the likelihood of response to PARPi monotherapy in the BRCA wild-type setting is low.

We also observed differential expression of genes in the MAPK and JAK/STAT pathways, which may have contributed additional mechanisms to the sensitivity of patients to the treatment regimen. These pathways are downstream of EGFR and also have been shown to cross-talk with DNA repair pathways [41–44]. In addition to DNA repair, the JAK/STAT pathway has also been shown to affect the expression of genes controlling immune signaling, including PD-L1 [45], as well as modulate the stemness of TNBC cells [46]. A recent phase 2 study of ruxolitinib monotherapy in TNBC has been reported [47], and despite on-target inhibition, the study did not meet its efficacy endpoint.

Although the patients included in this study were without germline mutation in BRCA1 or BRCA2, a BRCAness signature in HR repair mimicking BRCA1 or BRCA2 loss was seen in all 11 core biopsy samples collected prior to treatment. However, not all 11 patients responded to PARP inhibition. Nevertheless, significant differences in the DNA repair genes BRCA1, CRYAB, and CKB all showed downregulation in PR compared to PD samples. Clearly, more work is needed to uncover other biomarkers of PARP inhibitor sensitivity beyond HR mutations.

The majority of the patients enrolled in our trial had the basal subtype of TNBC, as expected. One of these patients (009) had a rapid response leading to her cancer becoming resectable (Fig. 3). At the time of this manuscript, she remains disease free 5 years after surgery and radiation therapy. Her pre-treatment biopsy showed undetectable BRCA1 mRNA, which could explain the dramatic response. However, the other responder (002) was classified to have HER2-enriched breast cancer from her biopsy. Interestingly, the response observed in this patient could be due to her low BRCA1 gene expression. Two other PD tissue samples had a similarly low BRCA1 expression level (basal 007, luminal A 008), indicating BRCA1 expression may not be the only indicator of PARP inhibitor sensitivity. Alternatively, her dramatic response could be due to the suppression of the HER2-PARP-NF-kB axis by PARP inhibition that we previously reported, which showed HER2+ breast cancer sensitivity to PARP inhibition independent of DNA repair [14, 48, 49].

The CRYAB (Crystallin αβ) and CKB (creatine kinase B) also showed downregulation in PR vs PD samples. Crystallin αβ is a structural protein of the eye lens that maintains transparency, but is also expressed in epithelial tumors. It is part of the heat shock family of proteins induced in the presence of cellular stress such as DNA damage to prevent apoptosis by inhibiting the cleavage of caspase-3 [50]. As the most abundant gene transcript present in early active multiple sclerosis legions, it is also a negative regulator acting as a brake on several inflammatory pathways in the immune and central nervous system, which could also connect the DDR with the immune system [51]. CKB is a cytosolic isoform of creatine kinase (brain type) and is upregulated in a variety of cancers. CKB enables cells to meet metabolic demands in hypoxic conditions and may play a role in cellular response to metabolic stress. In ovarian cancer, knockdown of this gene inhibited cancer cell proliferation and induced apoptosis, especially under hypoxia and hypoglycemic conditions, which induces a tumor-suppressive metabolic state with decreased glycolysis and elevated mitochondrial activity [52]. Deletion or inhibition of CKB alleviated bone loss during osteoclastogenesis, while hypoxia augments inflammation [53]. Interestingly, PARP1 directly binds to the CKB promoter region in this process, negatively regulating its transcription [54].

## Conclusions

The regimen of lapatinib and veliparib was well-tolerated with manageable toxicities for the combination of PARP inhibitors with EGFR/HER inhibitors. Exploratory biomarker analysis suggests potential involvement of immune response in the effect of the combination. Multiple ongoing clinical trials are currently evaluating combinations of immunotherapy with PARP inhibitors based on the principle that defective DNA repair leads to neoantigen expression; these studies are mostly limited in patients with germline or somatic BRCA1/2 mutations. By uncovering a synthetic interaction between EGFR signaling and DNA homologous recombination in BRCA wild-type TNBC and given the favorable toxicity profile of the veliparib–lapatinib combination, our results suggest that investigating this principle in BRCA wild-type TNBC may undercover new mechanisms of action. Because BRCA wild-type TNBC constitutes the majority of TNBC in practice (~ 75–85%) [6, 9, 10], further investigations of this combination in a larger trial with tissue analysis of biomarkers are warranted.

## Supplementary Information

**Additional file 1: Supplementary Figure S1.** Lapatinib concentration-time results from cycle 1 (triangles) and cycle 2 (circles). Vertical lines are standard error bars. **Supplementary Figure S2.** Plots of the Breast360 cancer signatures for each patient. PR are a) 002, b) 009; PD are c) 004, d) 005, e) 007, f) 008, g) 010, h) 013, i) 014, j) 022, k) 023. Inner circle: basal (red), her2-enhanced (pink), Lum B (lt. blue), Lum A (blue), TIS (green). Outer circle: tumor state (Claudin-low, differentiation), hormonal biology (AR, ER, ERBB2, ESR1, FOXA1, PGR), mutational susceptibility (BRCAness, HRD, p53 status), immune activity (CD8 + T cells, cytotoxicity), inhibition from tumor (TGFB, PDL1, IDO1, B7H3), inhibition from immune system (Treg, TIGIT, PDL2, Inflammation chemokines), tumor sensitivity (APM, apoptosis, proliferation), access to tumor (endothelial, stroma). **Supplementary Table S1.** Pharmacokinetic parameters for lapatinib during cycle 2 (steady-state). Abbreviations: AUC, area under the curve; Cmax, maximum concentration; CV, coefficient variation; Tmax time to maximum serum concentration; Elimination half-life, T_1/2_. **Supplementary Table S2.** Site of tissue biopsy and prior systemic treatments. **Supplementary Table 3.** Top Canonical Pathways.

**Additional file 2.** Independent Pathway Analysis details. This spreadsheet contains the data results from IPA.

**Additional file 3.** Independent Pathway Analysis. This file contains the major pathways found from IPA.

## Data Availability

All data generated or analyzed during the study are included in this published article [and its supplementary information files].
