# Supplementary material for: An open-label, pilot study of veliparib and lapatinib in patients with metastatic, triple-negative breast cancer
Source: Breast Cancer Res. 2021 Mar 4;23:30. doi: 10.1186/s13058-021-01408-9 (PMC7934554; doi:10.1186/s13058-021-01408-9)
Supplement: Supplementary file 1 — Additional file 1: Supplementary Figure S1. Lapatinib concentration-time results from cycle 1 (triangles) and cycle 2 (circles). Vertical lines are standard error bars. Supplementary Figure S2. Plots of the Breast360 cancer signatures for each patient. PR are a) 002, b) 009; PD are c) 004, d) 005, e) 007, f) 008, g) 010, h) 013, i) 014, j) 022, k) 023. Inner circle: basal (red), her2-enhanced (pink), Lum B (lt. blue), Lum A (blue), TIS (green). Outer circle: tumor state (Claudin-low, differentiation), hormonal biology (AR, ER, ERBB2, ESR1, FOXA1, PGR), mutational susceptibility (BRCAness, HRD, p53 status), immune activity (CD8 + T cells, cytotoxicity), inhibition from tumor (TGFB, PDL1, IDO1, B7H3), inhibition from immune system (Treg, TIGIT, PDL2, Inflammation chemokines), tumor sensitivity (APM, apoptosis, proliferation), access to tumor (endothelial, stroma). Supplementary Table S1. Pharmacokinetic parameters for lapatinib during cycle 2 (steady-state). Abbreviations: AUC, area under the curve; Cmax, maximum concentration; CV, coefficient variation; Tmax time to maximum serum concentration; Elimination half-life, T1/2. Supplementary Table S2. Site of tissue biopsy and prior systemic treatments. Supplementary Table 3. Top Canonical Pathways. [file 13058_2021_1408_MOESM1_ESM.docx]

**Supplementary Figure** **S1**. Lapatinib concentration-time results from cycle 1 (triangles) and cycle 2 (circles). Vertical lines are standard error bars.


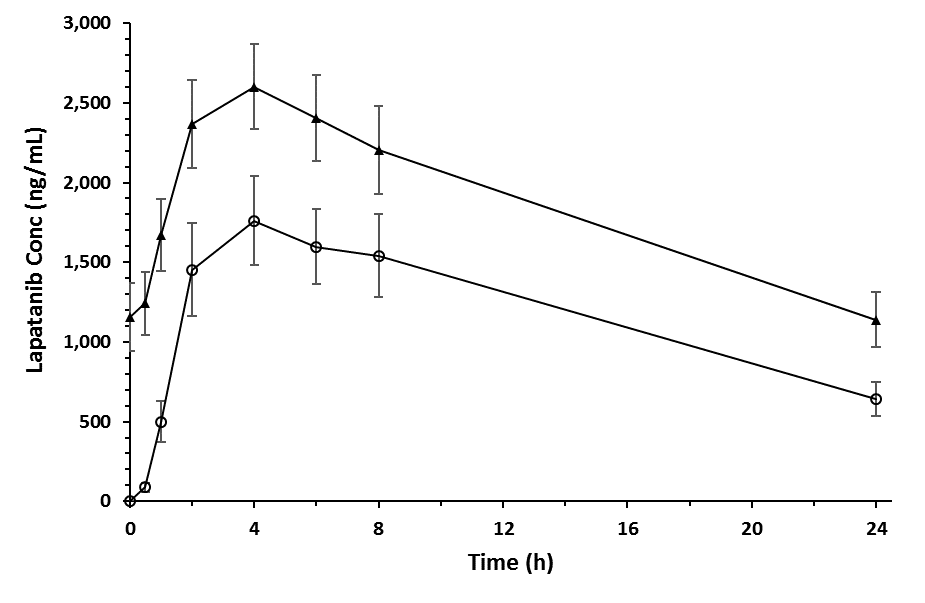


**Supplementary Figure S2.**  Plots of the Breast360 cancer signatures for each patient. PR are a) 002, b) 009; PD are c) 004, d) 005, e) 007, f) 008, g) 010, h) 013, i) 014, j) 022, k) 023. Inner circle: basal (red), her2-enhanced (pink), Lum B (lt. blue), Lum A (blue), TIS (green). Outer circle: tumor state (Claudin-low, differentiation), hormonal biology (AR, ER, ERBB2, ESR1, FOXA1, PGR), mutational susceptibility (BRCAness, HRD, p53 status), immune activity (CD8+T cells, cytotoxicity), inhibition from tumor (TGFB, PDL1, IDO1, B7H3), inhibition from immune system (Treg, TIGIT, PDL2, Inflammation chemokines), tumor sensitivity (APM, apoptosis, proliferation), access to tumor (endothelial, stroma).

**a)**

**b)**

**c)**

**d)**

**e)**

**f)**

**g)**

**h)**

**i)**

**j)**

**k)**

**Supplementary Table S1**. Pharmacokinetic parameters for lapatinib during cycle 2 (steady-state). Abbreviations: AUC, area under the curve; Cmax, maximum concentration; CV, coefficient variation; Tmax time to maximum serum concentration; Elimination half-life, T_1/2_.

| **Parameter** | **Mean** | **CV%** |
| --- | --- | --- |
| AUC_24_ (mgxh/L) | 44.1 | 46.11 |
| Cmax (mg/L) | 2.84 | 37.35 |
| T_1/2_ (hr) | 17.08 | 58.77 |
| Tmax (hr) | 4.18 | 48.32 |

**Supplementary Table S2.** Site of tissue biopsy and prior systemic treatments.

| **Patient Number** | **Biopsy Site** | **Prior Systemic Treatments Received for TNBC** |
| --- | --- | --- |
| 002 | Right Cervical lymph node | *None |
| 004 | Lung | Doxorubicin+cyclophosphamide  Anti-PD-L1 + Paclitaxel protein bound (clinical trial) |
| 005 | Skin-chest wall | *Paclitaxel+anti-HER3 monoclonal Antibody (clinical trial) |
| 007 | Skin-chest wall | *None |
| 008 | Skin-breast | Ganitumab (*neoadjuvant clinical trial) |
| 009 | Skin-breast | *None |
| 010 | Liver | Doxorubicin+ cyclophosphamide  Paclitaxel |
| 013 | Breast | Abraxane  Doxorubicin liposomal |
| 014 | Axillary lymph node | Doxorubicin+ cyclophosphamide  Paclitaxel |
| 022 | Breast | Docetaxel  Doxorubicin liposomal |
| 023 | Skin-chest wall | *Capecitabine  Paclitaxel protein bound +placebo/atezolizumab (clinical trial) |

- Received neoadjuvant and/or adjuvant anthracycline-based chemotherapy and taxanes.

**Supplementary Table 3.** Top Canonical Pathways

| **Pathway Name** | **p-value** | **Overlap** |
| --- | --- | --- |
| Th1 | 1.71E-16 | 9.90%, 12/121 |
| Th1 and Th2 Activation | 1.17E-14 | 7.00%, 12/171 |
| T Cell Exhaustion Signaling | 5.58E-13 | 6.30%, 11/175 |
| Neuroinflammation Signaling | 9.35E-12 | 4.00%, 12/300 |
| MSP-RON Signaling In Macrophages | 9.89E-12 | 8.00%, 9/113 |
