# Supplementary material for: An open-label, pilot study of veliparib and lapatinib in patients with metastatic, triple-negative breast cancer
Source: Breast Cancer Res. 2021 Mar 4;23:30. doi: 10.1186/s13058-021-01408-9 (PMC7934554; doi:10.1186/s13058-021-01408-9)
Supplement: Supplementary file 3 — Additional file 3. Independent Pathway Analysis. This file contains the major pathways found from IPA. [file 13058_2021_1408_MOESM3_ESM.pdf]

INGENUITY
PATHWAY ANALYSIS

Analysis Name: DEresults-New.AnnotationPR
Analysis Creation Date: 2020-12-04
Build version: exported
Content version: 57662101 (Release Date: 2020-09-15)

Experiment Metadata

Name	Value

Analysis Settings

Reference set: Ingenuity Knowledge Base (Genes Only)
Relationship to include: Direct and Indirect
Includes Endogenous Chemicals
Optional Analyses: My Pathways My List

Filter Summary:
Consider only molecules and/or relationships where
(species = Mouse OR Uncategorized OR Rat OR Human) AND
(confidence = Experimentally Observed) AND
(tissues/cell lines = A498 OR SNB-75 OR Neurons not otherwise specified OR Mature monocyte-derived dendritic cells OR Other Osteosarcoma Cell Lines OR BT-474 OR HCC-2998 OR Thalamus OR Mesenchymal stem cells OR Stomach OR Intraepithelial T lymphocytes OR ACHN OR Other Cervical cancer cell line OR Teratocarcinoma Cell Lines not otherwise specified OR Smooth muscle cells not otherwise specified OR

(c) 2000-2020 QIAGEN. All rights reserved.

Other Epithelial cells OR Dendritic cells not otherwise specified OR Other Tissues and Primary Cells OR Effector T cells OR Other Lymphocytes OR Effector memory cytotoxic T cells OR Uterus OR Keratinocytes OR Other Bone marrow cells OR Ovary OR Peripheral blood lymphocytes OR Tissues and Primary Cells not otherwise specified OR NIH/3T3 cells OR BDCA-3+ dendritic cells OR Megakaryocytes OR Effector memory helper T cells OR U87MG OR NCI-ADR-RES OR NCI-H522 OR U266 OR Other B lymphocytes OR Activated helper T cells OR MDA-MB-231 OR Peritoneal macrophages OR Microvascular endothelial cells OR Pre-B lymphocytes OR HEL OR Salivary Gland OR Esophagus OR Fibroblast cell lines not otherwise specified OR Prostate Cancer Cell Lines not otherwise specified OR Stem cells not otherwise specified OR Cerebral Ventricles OR Vascular smooth muscle cells OR Pituitary Gland OR Other Kidney cell lines OR Cells not otherwise specified OR Pancreatic Cancer Cell Lines not otherwise specified OR Langerhans cells OR Striatum OR Other Cells OR Eosinophils OR MDA-MB-435 OR Other Hepatoma Cell Lines OR Other Kidney Cancer Cell Lines OR Cerebral Cortex OR HL-60 OR NK cells not otherwise specified OR Plasma cells OR Mononuclear leukocytes not otherwise specified OR Other Neurons OR Vd2 Gamma-delta T cells OR Central memory cytotoxic T cells OR Effector memory RA+ cytotoxic T cells OR Other Neuroblastoma Cell Lines OR Other T lymphocytes OR Activated CD56dim NK cells OR Other Pheochromocytoma cell lines OR UACC-62 OR Hematopoietic progenitor cells OR Neuroblastoma Cell Lines not otherwise specified OR CNS Cell Lines not otherwise specified OR Hepatoma Cell Lines not otherwise specified OR Purkinje cells OR BA/F3 OR Amygdala OR M14 OR NT2/D1 OR Leukemia Cell Lines not otherwise specified OR Forestomach OR THP-1 OR Activated CD56bright NK cells OR CCRF-CEM OR DU-145 OR PC-12 cells OR RBL-2H3 OR K-562 OR CD4+ T-lymphocytes OR White Matter OR CAKI-1 OR INS-1 OR Natural T-regulatory cells OR Other Peripheral blood leukocytes OR A375 OR PC-3 OR Cartilage Tissue OR Skeletal Muscle OR A549-ATCC OR Parietal Lobe OR HOP-92 OR Th2 cells OR Neutrophils OR Chondrocytes OR Sciatic Nerve OR H460 OR Crypt OR Calvaria OR Cytotoxic T cells OR Memory B cells OR NB4 OR Activated Vd2 Gamma-delta T cells OR Astrocytes OR Olfactory Bulb OR SR OR BT-549 OR HeLa OR Other Myeloma Cell Lines OR Brainstem OR Cerebellum OR Other Lung Cancer Cell Lines OR Trachea OR Swiss 3T3 cells OR Osteosarcoma Cell Lines not otherwise specified OR Cervical cancer cell line not otherwise specified OR MALME-3M OR Other Endothelial cells OR Choroid Plexus OR Naive helper T cells OR Other Macrophage Cancer Cell Lines OR Oocytes OR SN12C OR Kidney cell lines not otherwise specified OR T47-D OR Other Melanoma Cell Lines OR Testis OR Cortical neurons OR Monocytes not otherwise specified OR Pro-B lymphocytes OR Prostate Gland OR HMC-1 OR Other Fibroblast cell lines OR UO-31 OR Cardiomyocytes OR Monocyte-derived dendritic cells not otherwise specified OR SW-480 OR Bone marrow cells not otherwise specified OR T lymphocytes not otherwise specified OR Other Lymphoma Cell Lines OR SK-MEL-28 OR LNCaP cells OR Medulla Oblongata OR OVCAR-5 OR PANC-1 OR Other Smooth muscle cells OR Beta islet cells OR Vd1 Gamma-delta T cells OR J-774A.1 OR KM-12 OR CD56bright NK cells OR Other NK cells OR SW-620 OR IGROV1 OR OVCAR-3 OR Other Nervous System OR Peripheral blood monocytes OR Other Leukemia Cell Lines OR Other Teratocarcinoma Cell Lines OR Activated Vd1 Gamma-delta T cells OR SF-539 OR Ovarian Cancer Cell Lines not otherwise specified OR Brain OR Heart OR Hepatocytes OR Organ Systems not otherwise specified OR SF-295 OR Pheochromocytoma cell lines not otherwise specified OR RAW 264.7 OR Lung Cancer Cell Lines not otherwise specified OR

(c) 2000-2020 QIAGEN. All rights reserved.

SK-N-SH OR Skin OR Lung OR HS 578T OR Lymphocytes not otherwise specified OR Lymphoma Cell Lines not otherwise specified OR Other Ovarian Cancer Cell Lines OR Bone marrow-derived dendritic cells OR Putamen OR 3T3-L1 cells OR Kidney Cancer Cell Lines not otherwise specified OR HUVEC cells OR B lymphocytes not otherwise specified OR Min6 OR Adipocytes OR MDA-N OR Hypothalamus OR Other Mononuclear leukocytes OR Embryonic stem cells OR Blood platelets OR Th17 cells OR Epithelial cells not otherwise specified OR Granule Cell Layer OR HepG2 OR Peripheral blood leukocytes not otherwise specified OR Caudate Nucleus OR Other Immune cells OR Other Monocytes OR Melanocytes OR Other CNS Cell Lines OR Other Cell Line OR RKO OR Dorsal Root Ganglion OR Gray Matter OR Bone marrow-derived macrophages OR Macrophages not otherwise specified OR Other Memory T lymphocytes OR OVCAR-4 OR Granulosa cells OR HCT-116 OR OVCAR-8 OR SF-268 OR Cos-7 cells OR Splenocytes OR Lens OR Small Intestine OR Kidney OR HuH7 OR J774 OR HOP-62 OR Jurkat OR U251 OR Adipose OR Other Granulocytes OR Other Monocyte-derived dendritic cells OR MDA-MB-361 OR Ventricular Zone OR Monocyte-derived macrophage OR Immature monocyte-derived dendritic cells OR Adrenal Gland OR HT29 OR U2OS OR Hippocampus OR NCI-H332M OR Other Colon Cancer Cell Lines OR Mammary Gland OR Spinal Cord OR Nervous System not otherwise specified OR NCI-H226 OR WEHI-231 OR Bladder OR Other Breast Cancer Cell Lines OR Granule cells OR Liver OR A2780 OR Large Intestine OR Placenta OR Melanoma Cell Lines not otherwise specified OR Myeloma Cell Lines not otherwise specified OR EKVX OR MOLT-4 OR PBMCs OR Trigeminal Ganglion OR BDCA-1+ dendritic cells OR Other Dendritic cells OR Corpus Callosum OR Cornea OR Memory T lymphocytes not otherwise specified OR LOX IMVI OR Naive B cells OR Pyramidal neurons OR COLO205 OR Dermis OR Colon Cancer Cell Lines not otherwise specified OR CD34+ cells OR MCF7 OR Central memory helper T cells OR Breast Cancer Cell Lines not otherwise specified OR SK-MEL-2 OR Other Organ Systems OR Stromal cells OR Immune cell lines not otherwise specified OR Pancreas OR Sertoli cells OR SK-OV-3 OR Microglia OR Fibroblasts OR Th1 cells OR MDA-MB-468 OR Lymph node OR UACC-257 OR Thymocytes OR Macrophage Cancer Cell Lines not otherwise specified OR Hep3B OR MEF cells OR Other Immune cell lines OR SK-MEL-5 OR NCI-H23 OR Spleen OR 293 cells OR Cell Line not otherwise specified OR Granulocytes not otherwise specified OR Caco2 cells OR Epidermis OR Other Stem cells OR Thymus OR CD56dim NK cells OR Mast cells OR 786-0 OR U937 OR Plasmacytoid dendritic cells OR Osteoblasts OR Substantia Nigra OR Nucleus Accumbens OR Immune cells not otherwise specified OR Retina OR P19 OR Murine NKT cells OR TK-10 OR Other Macrophages OR Myeloid dendritic cells OR Subventricular Zone OR Thyroid Gland OR RXF-393 OR MG-63 OR Other Pancreatic Cancer Cell Lines OR Smooth Muscle OR Endothelial cells not otherwise specified OR HCT-15 OR Other Prostate Cancer Cell Lines OR RPMI-8266) AND

(mol. types = biologic drug OR canonical pathway OR chemical - endogenous mammalian OR chemical - endogenous non-mammalian OR chemical - kinase inhibitor OR chemical - other OR chemical - protease inhibitor OR chemical drug OR chemical reagent OR chemical toxicant OR complex OR cytokine OR disease OR enzyme OR function OR fusion gene/product OR G-protein coupled receptor OR group OR growth factor OR ion channel OR kinase OR ligand-dependent nuclear receptor OR mature microRNA OR microRNA OR other OR peptidase OR phosphatase OR transcription regulator OR translation regulator OR transmembrane receptor OR transporter) AND

(c) 2000-2020 QIAGEN. All rights reserved.

INGENUITY PATHWAY ANALYSIS

(data sources = An Open Access Database of Genome-wide Association Results OR BIND OR BioGRID OR Catalogue Of Somatic Mutations In Cancer (COSMIC) OR Chemical Carcinogenesis Research Information System (CCRIS) OR ClinicalTrials.gov OR ClinVar OR Cognia OR DIP OR DrugBank OR Gene Ontology (GO) OR GVK Biosciences OR Hazardous Substances Data Bank (HSDB) OR HumanCyc OR Ingenuity Expert Findings OR Ingenuity ExpertAssist Findings OR IntAct OR Interactome studies OR MIPS OR miRBase OR miRecords OR Mouse Genome Database (MGD) OR Obesity Gene Map Database OR Online Mendelian Inheritance in Man (OMIM) OR TarBase OR TargetScan Human)

Top Canonical Pathways

Name	p-value	Overlap
**Th1 Pathway**	1.71E-16	9.9 % 12/121
**Th1 and Th2 Activation Pathway**	1.17E-14	7.0 % 12/171
**T Cell Exhaustion Signaling Pathway**	5.58E-13	6.3 % 11/175
**Neuroinflammation Signaling Pathway**	9.35E-12	4.0 % 12/300
**MSP-RON Signaling In Macrophages Pathway**	9.89E-12	8.0 % 9/113

Top Upstream Regulators

Upstream Regulators

Name	p-value	Predicted Activation
**progesterone**	7.26E-13	
**IL27**	3.48E-12	Activated
**lipopolysaccharide**	4.59E-12	Activated

(c) 2000-2020 QIAGEN. All rights reserved.

IL1B	2.06E-11	Activated
beta-estradiol	4.25E-11	

Causal Network

Name	p-value	Predicted Activation
IL27	4.06E-19	Activated
TREM2	4.45E-17	
EBI3	4.78E-17	Activated
Eotaxin	9.11E-16	
Ccl2	1.72E-15	

Top Diseases and Bio Functions

Diseases and Disorders

Name	p-value range	# Molecules
Inflammatory Response	6.15E-05 - 7.37E-17	40
Organismal Injury and Abnormalities	6.15E-05 - 7.37E-17	56
Connective Tissue Disorders	3.78E-05 - 1.97E-14	34
Inflammatory Disease	4.69E-05 - 1.97E-14	37
Skeletal and Muscular Disorders	3.78E-05 - 1.97E-14	34

Molecular and Cellular Functions

(c) 2000-2020 QIAGEN. All rights reserved.

INGENUITY PATHWAY ANALYSIS

Name	p-value range	# Molecules
**Cell-To-Cell Signaling and Interaction**	6.15E-05 - 1.27E-16	33
**Cellular Function and Maintenance**	5.57E-05 - 3.48E-15	34
**Cellular Movement**	6.15E-05 - 1.41E-12	30
**Cellular Development**	5.57E-05 - 4.57E-12	40
**Cellular Growth and Proliferation**	5.57E-05 - 4.57E-12	42

Physiological System Development and Function

Name	p-value range	# Molecules
**Hematological System Development and Function**	6.15E-05 - 1.27E-16	35
**Immune Cell Trafficking**	5.79E-05 - 1.27E-16	27
**Cell-mediated Immune Response**	5.57E-05 - 5.08E-13	24
**Lymphoid Tissue Structure and Development**	5.57E-05 - 9.23E-13	29
**Tissue Morphology**	6.15E-05 - 9.23E-13	32

Top Tox Functions

Assays: Clinical Chemistry and Hematology

Name	p-value range	# Molecules
**Increased Levels of Alkaline Phosphatase**	1.41E-02 - 1.41E-02	2
**Increased Levels of LDH**	2.97E-02 - 2.97E-02	1

(c) 2000-2020 QIAGEN. All rights reserved.

**Increased Levels of Creatinine**	1.42E-01 - 1.42E-01	1
**Increased Levels of Red Blood Cells**	2.40E-01 - 2.40E-01	1

Cardiotoxicity

Name	p-value range	# Molecules
**Cardiac Dysfunction**	2.44E-01 - 1.26E-05	7
**Cardiac Enlargement**	2.44E-01 - 3.33E-05	9
**Cardiac Inflammation**	1.14E-03 - 2.39E-04	3
**Heart Failure**	6.09E-02 - 4.09E-04	6
**Cardiac Proliferation**	4.18E-02 - 4.46E-04	4

Hepatotoxicity

Name	p-value range	# Molecules
**Liver Fibrosis**	9.65E-03 - 4.68E-09	12
**Liver Damage**	9.99E-03 - 5.11E-09	9
**Liver Necrosis/Cell Death**	5.38E-02 - 2.84E-07	8
**Liver Inflammation/Hepatitis**	1.86E-01 - 3.51E-07	10
**Liver Cirrhosis**	7.59E-06 - 3.02E-06	8

Nephrotoxicity

Name	p-value range	# Molecules

(c) 2000-2020 QIAGEN. All rights reserved.

**Renal Necrosis/Cell Death**	6.32E-02 - 7.07E-05	7
**Glomerular Injury**	1.53E-01 - 1.71E-04	4
**Renal Inflammation**	9.11E-02 - 1.71E-04	5
**Renal Nephritis**	9.11E-02 - 1.71E-04	5
**Renal Damage**	2.17E-01 - 1.46E-03	5

Top Regulator Effect Networks

ID	Regulators	Disease & Functions	Consistency Score
**1**	AGT,cytokine,IFNA2,Ifnar,IFNG,IL15,IL1B,IL2,IL21 (+6 more)	Activation of monocytes (+21 more)	72.569
**2**	AGT,RETNLB,TLR4,TNNI3	Activation of leukocytes (+12 more)	39.799
**3**	Ifnar	Infection of mammalia,Leukopoiesis (+1 more)	4.5
**4**	EBI3	Activation of leukocytes	-4.899
**5**	HIF1A	Chemotaxis of macrophages	-7.0

Top Networks

ID	Associated Network Functions	Score
**1**	Cell-To-Cell Signaling and Interaction, Hematological System Development and Function, Immune Cell Trafficking	27

(c) 2000-2020 QIAGEN. All rights reserved.

**2**	Inflammatory Response, Organismal Injury and Abnormalities, Hematological Disease	22
**3**	Gastrointestinal Disease, Hepatic System Disease, Liver Cholestasis	17
**4**	Cell-To-Cell Signaling and Interaction, Cellular Movement, Hematological System Development and Function	17
**5**	Cell Morphology, Immunological Disease, Cell Death and Survival	13

Top Tox Lists

Name	p-value	Overlap
**Hepatic Fibrosis**	2.06E-07	2.6 % 9/350
**Liver Necrosis/Cell Death**	1.47E-06	2.5 % 8/326
**Increases Liver Damage**	3.84E-06	5.3 % 5/94
**Xenobiotic Metabolism Signaling**	2.36E-04	1.7 % 6/349
**NRF2-mediated Oxidative Stress Response**	3.31E-04	2.1 % 5/239

Top My Lists

(c) 2000-2020 QIAGEN. All rights reserved.

INGENUITY PATHWAY ANALYSIS

Top My Pathways

Top Analysis-Ready Molecules

Expr Log Ratio

Molecules	Expr. Value	Chart
GJB2	↑ 3.210	
HSPA2	↑ 2.830	
CCL5	↑ 2.350	
HLA-DOB	↑ 2.310	
LFNG	↑ 2.260	
CCL8	↑ 2.180	
GZMA	↑ 2.050	
PLA2G3	↑ 2.030	
HGF	↑ 2.020	
LAG3	↑ 1.970	

Expr Log Ratio

Molecules	Expr. Value	Chart
GABRP	↓ -4.780	
MMP7	↓ -4.260	
PTCH1	↓ -4.170	
PROM1	↓ -4.080	

(c) 2000-2020 QIAGEN. All rights reserved.

INGENUITY PATHWAY ANALYSIS

CALML5	↓	-4.030
HBB	↓	-3.980
RASGRF1	↓	-3.350
COL11A1	↓	-3.050
CKB	↓	-3.030
CRYAB	↓	-2.880

(c) 2000-2020 QIAGEN. All rights reserved.

INGENUITY PATHWAY ANALYSIS
